# Effect of Plyometric Training on Jumping, Sprinting and Change of Direction Speed in Child Female Athletes

**DOI:** 10.3390/sports7050116

**Published:** 2019-05-17

**Authors:** Gregory C. Bogdanis, Olyvia Donti, Athanasia Papia, Anastasia Donti, Nikolaos Apostolidis, William A. Sands

**Affiliations:** 1 Sports Performance Laboratory, School of Physical Education & Sport Science, National and Kapodistrian University of Athens, 17237 Athens, Greece; apapia@phed.uoa.gr (A.P.); adonti@phed.uoa.gr (A.D.); napost@phed.uoa.gr (N.A.); 2 United States Ski and Snowboard Association; Salt Lake City, UT 84109, USA; wmasands@hotmail.com

**Keywords:** muscle power, stretch–shortening cycle, children, gymnastics

## Abstract

Background: This study examined the effects of 8 weeks of plyometric training on jumping, sprinting, and change of direction (COD) performance. Methods: Fifty female 7–9-year-old gymnasts were randomly assigned to a plyometric training group (PG; *n* = 33), that performed supplementary plyometric training twice per week, and a control group (CG; *n* = 17) that continued regular training. The following tests were performed before and after the intervention: 10 and 20 m sprints, 5 + 5 m and 10 + 10 m COD tests, one-leg and two-leg countermovement jump (CMJ), drop jump (DJ), squat jump (SJ), and standing long jump (SLJ). Results: Only a main effect for time was found for all jumping performance parameters (*p* = 0.001). However, the improvement of one- and two-leg CMJ in PG had a greater effect size than CG (0.72 and 0.67 vs. 0.34 and 0.18, respectively). Group × time interactions were found for 10 and 20 m sprint tests (*p* = 0.018 and *p* = 0.011, respectively) and for 10 + 10 m COD (*p* = 0.008) with the post hoc test showing improvement only for the PG (*p* = 0.001, 0.001, and 0.003 and d = 1.1, 1.14, and 0.6, respectively). Conclusions: Supplementary plyometric training increased sprint and COD performance more than regular gymnastics training, while jumping performance was equally improved in both groups.

## 1. Introduction

Leg muscle power and sprinting ability are important performance parameters in several youth sports [1,2]. The effects of training on muscular power and jumping ability are more pronounced in adolescence than childhood, with boys improving more than girls [3]. Adaptations to sprint training are more variable, showing an initial spurt during the ages of 5–9 years, which is followed by a second period of rapid improvement during puberty and beyond [2,4]. A more complex fitness component associated with straight line sprinting, muscular power, and motor coordination, is the change of direction (COD) speed [5]. Ιn most youth development models, drills including short sprints, accelerations, decelerations, and COD may be used as training tools, from a very young age, to develop general athleticism as well as physical qualities, such as speed and muscle power [1,2,6].

Plyometric training is effective in increasing sprinting and jumping ability, reactive strength, and COD in prepubertal athletes [7,8]. Age, maturation, and task complexity influence plyometric training effectiveness [9,10]. For example, Moran et al. [11] reported a greater increase in vertical jumping following plyometric training in males before and after peak height velocity (PHV) compared to mid-PHV (d ˃ 0.90, and d = 0.47, respectively), while Asadi et al. [12] found lower adaptive responses in COD abilities, in male participants before PHV (d = 0.68), compared with mid-and post-PHV (d = 0.95 and 0.99, respectively). However, most studies have examined male youth athletes, while females may respond differently due to the gender-specific effects of maturation on body composition and adaptability to training [3,13]. Recently, Moran et al. [14] reported that plyometric training induced small effects (d = 0.57) on jumping performance in female youth (8–18 years), while there is limited evidence on the degree to which prepubescent girls adapt to plyometric training. 

The fundamental locomotion skills of jumping, leaping, hopping, and running may be significantly improved during middle childhood (7–10 years), while it has been recommended that children may start resistance training with their own body weight as early as they are able to follow instructions and safety rules (i.e., 7–8 years of age) [1,15,16]. However, age- and sport-specific information is limited regarding the effects of plyometric training on speed and power indices in very young athletes, and especially females, although in some sports like gymnastics, children are training systematically from a very early age [3,6]. Young gymnasts are required to execute complex technical skills that require high power output acquired from a young age [6]. Thus, the purpose of this study was to examine the effects of 8 weeks of plyometric training on jumping performance, sprinting, and COD in 7–9 years old female gymnasts. It was hypothesized that supplementary plyometric training would improve these abilities more than gymnastics training alone.

## 2. Materials and Methods

### 2.1. Participants

Power calculations indicated a sample of 12 participants would be needed to detect an effect size (ES) of 0.5, obtained from the minimum ES reported in the meta-analysis of Moran et al. [11] for plyometric training in pre-PHV boys (within–between ANOVA power = 0.80, alpha = 0.05, correlation between repeated measures r = 0.5; G-Power 3.1.9.2). Gymnast ages ranged from 7 to 9 years, and they were recruited from the same gymnastics club and trained with the same coach in three groups of 16–19 athletes each. None of the participants had performed systematic plyometric training in the past. All participants were injury-free six months prior to the study start and no gymnast was injured during the study. Since the minimum sample size was 12, it was decided that two of the groups would be randomly allocated to the plyometric intervention (*n* = 16 and *n* = 19) while the other group acted as a control (*n* = 18). Of these, two gymnasts from the training group and one athlete from the control group did not perform all preliminary tests and visits, and were excluded from the study. In total, fifty female ‘Gymnastics for All’ gymnasts participated in this study forming the plyometric group (PG; *n* = 33) and the control group (CG; *n* = 17). No athlete dropped out during the course of the intervention.

Anthropometric and maturity characteristics of the participants are shown in Table 1. All gymnasts trained 3 days per week, (Monday, Wednesday, Friday) for 90 min each day, and competed in the national championships. Maturity offset was calculated at the study beginning and end, according to the prediction equation of Mirwald et al. [17]. Based on the chronological age and maturity offset (−4.9 ± 0.4 years from peak height velocity—PHV) the participants of the PG and the CG were characterized as prepubertal. Prior to the study, the athletes and their parents were fully informed about the purpose and risks of this study. Written parental consent was obtained for each participant. Procedures were approved by the Institutional Ethics Review Committee (reference number: 1000/30-09-2016) and complied with the Code of Ethics of the World Medical Association (Helsinki declaration of 1964, as revised in 2013).

### 2.2. Study Design

The effects of 8 weeks of plyometric training, in addition to regular gymnastics training, on jumping, sprinting, and COD were examined using a repeated measures design. This 8-week period was chosen based on the duration of a typical mesocycle. The study took place during the gymnastics preseason (October to December). All gymnasts underwent the following tests at baseline, and after 8 weeks of training: 10 and 20 m linear sprint test, 5 + 5 m and 10 + 10 m COD sprint tests with a 180° turn, one-leg and two-leg countermovement jump (CMJ), drop jump (DJ), squat jump (SJ), and standing long jump (SLJ). The additional plyometric program was performed during non-consecutive days (Monday, Wednesday) before the main training and lasted ~27 min (total of 16 sessions). In the two weeks preceding baseline testing, four familiarization sessions were held to get participants familiarized with the plyometric training and testing procedures and to calculate intra-class correlation coefficients (ICC) using a two-way mixed model.

### 2.3. Methodology

Body mass, (0.1 kg, Seca 700, Seca Ltd., Birmingham, UK) standing height and sitting height (0.1 cm, Charder HM-200P, Charder Electronic Co., Ltd., Taichung, Taiwan) were measured before and after training.

#### 2.3.1. Jumping Performance

Jump height was determined from flight time, using an Optojump system (Microgate, SRL, Bolzano, Italy). The validity and reliability of this system for measuring jump height have been previously reported [18]. Participants were instructed to perform maximum effort jumps. For the one- and two-leg CMJ, DJ, and SJ, participants were instructed to maintain their hands akimbo, to take off with the ankles and knees fully extended, and to land balanced on the same spot. During all jumping tests, gymnasts wore gymnastics shoes. For the one- and the two-leg CMJ, gymnasts were instructed to perform a countermovement until the knees bent to approximately 90°, and then immediately jump up. ICC for the two-leg CMJ was 0.94 (95% CI: 0.90–0.97) (SEM = 4.3%, MDC_90_ = 1.8 cm) and for the sum of the right and the left-leg CMJ it was 0.97 (95% CI: 0.94–0.98) (SEM = 5.1%, MDC_90_ = 1.7 cm).

For the DJ, gymnasts stepped horizontally off a 20 cm box on the gymnastics carpet and then immediately performed a maximal rebound vertical jump with minimal ground contact time (CT). The ICC for the DJ height was 0.93 (95% CI: 0.87–0.96) (SEM = 7.4%, MDC_90_ = 2.6 cm), while the ICC for CT was 0.85 (95% CI: 0.73–0.91) (SEM = 10.3%, MDC_90_ = 100 ms).

Reactive strength index (RSI) was determined during drop jump as the ratio between jump height and time spent in contact with the ground as follows [19]:

RSI = jump height (millimeters)/ground contact time (milliseconds).

The ICC for RSI was 0.86 (95% CI: 0.75–0.92) (SEM = 16.6%, MDC_90_ = 0.20 mm/ms).

For the SJ, participants started from a stationary semi-squat position (~90° knee flexion determined by a manual goniometer) and were instructed to jump as high as possible, without a countermovement. For each trial of the SLJ, the participants started behind a line on the ground, feet at shoulder width and hands neutral. On the command, participants executed a countermovement and then jumped maximally in the horizontal direction. Participants landed with both feet simultaneously and were not allowed to fall. Horizontal distance from the toes at the start to the landing at heel contact was used for statistical analysis. All trials were measured to the nearest 0.01 m. The ICC for the SJ was 0.89 (95% CI: 0.80–0.94) (SEM = 7.2%, MDC_90_ = 2.8 cm) and for the SLJ, 0.85 (95% CI: 0.74–0.92) (SEM = 5.5%, MDC_95_ = 14.0 cm).

#### 2.3.2. Sprint and COD Tests

Ten- and twenty-meter linear sprint performances were assessed electronically (Microgate, SARL, Bolzano, Italy). Participants were asked to stand in an upright stride stance with the preferred leg forward, 0.3 m before the first infrared photoelectric gate, which was placed 0.75 m above the ground to ensure it captured trunk movement and avoided false limb motion signals. The intraclass correlation coefficient for the 10 m sprint was 0.95 (95% CI: 0.90–0.97) (SEM = 2.1%, MDC_90_ = 0.14 s) and for the 20 m sprint ICC was 0.96 (95% CI: 0.91–0.98) (SEM = 1.9%, MDC_90_ = 0.21 s).

COD tests were performed over two distances: 5 + 5 m and 10 + 10 m with a 180° turn. Cones were placed at 0, 5, and 10 m on a gymnastics vault runway. Participants were instructed to accelerate as quickly as possible along the 5 m distance, pivot 180° around the cone, and return as quickly as possible to the starting line. The same procedure was repeated for the 10 + 10 m COD. The total time to run the COD tests was measured electronically (Microgate, SARL, Bolzano, Italy). The ICC for 5 + 5 m COD was 0.88 (95% CI: 0.78–0.93) (SEM = 2.6%, MDC_90_ = 0.23 s) and for the 10 + 10 m COD, it was 0.94 (95% CI: 0.89–0.97) (SEM = 1.6%, MDC_90_ = 0.22s).

All performance tests were completed in the same session, 48 h after the last training and following a 10 min standardized warm-up, including 5 min of light jogging, dynamic stretches, and 2 short accelerations. For the 10 and 20 m of linear sprint tests and the COD tests, athletes performed 2 trials for each distance interspersed by 1 min of rest and the best time was recorded for further analysis. Sprint and COD tests were separated by 3 min of passive rest. After that, and following 3 min of recovery, the gymnasts performed the jump tests. Two trials were performed for each jump and the average value was used for analysis. Trials were separated by 30 s and there was a 2 min rest between the different jump tests.

#### 2.3.3. Plyometric Training

At the start of the Monday and Wednesday training session, and immediately after the 10 min standardized warm-up, the athletes of the PG performed plyometric training while the athletes of the CG performed choreography movements. Following the plyometric program all gymnasts continued their regular gymnastics training. The plyometric program was designed to include two 4-week training blocks. Six plyometric exercises of progressive difficulty were performed in each training block according to the stages of plyometric load proposed by Lloyd et al. [20]. In each session, athletes performed two rounds of six exercises in a circuit form, with 30 s rest between exercises and 5 min of rest between rounds (Table 2). Plyometric exercises were performed on the surface of a gymnastics carpet with the gymnasts wearing gymnastics shoes. Plyometric training was supervised by an experienced coach and proper technique of movement was emphasized at every training and testing session. Also, an experienced coach recorded all the gymnastics elements with a plyometric component for the lower limbs (e.g., handsprings, round-off, vaulting) during all training sessions, to ensure that all participants received similar plyometric training load. In the first week of training gymnasts in both groups performed ~100 gymnastics elements with a plyometric component in each training session and increasing to ~130 gymnastics elements by study completion.

### 2.4. Statistical Analyses

Statistical analyses were carried out using SPSS (IBM SPSS Statistics, Version 22.0, IBM corporation, Armonk, New York, NY, USA). Data are presented as means and standard deviations. The 90% confidence intervals were also calculated for the mean differences reported. The normality of data distributions was checked using a Kolmogorov–Smirnov test. One-way analyses of variance (ANOVA) were applied to determine significant differences in baseline values between groups. A two-way ΑΝΟVA (group (plyometric/control) × time (pre/post-training)), with repeated measures on the time dimension, was conducted to examine the effect of plyometric training on all examined variables. When a significant main effect or interaction was observed (*p* < 0.05) a Tukey’s post hoc test was performed. Effect sizes (ES) for the ANOVA were determined by partial eta squared (η^2^). For pairwise comparisons, ES was determined by Cohen’s d. Magnitude-based inference (MBI) tests were performed, as described by Hopkins et al. [21]. The threshold value for the smallest worthwhile change was set in this study at 0.2 of the between subject standard deviation. The scale used to interpret the probabilities was as follows: 25–75%, possible; 75–95%, likely; 95–99%, very likely; ˃99.5%, most likely.

Additionally, the standard error of measurement (SEM) and the meaningful detectable change at 90% confidence interval (MDC_90_) were calculated. Statistical significance was set at *p* < 0.05.

## 3. Results

### 3.1. Anthropometric Measurement

There were no statistical differences in age, training experience, maturity offset, and anthropometric characteristics between the PG and the CG at baseline (Table 1). After training, there was a statistically significant increase in standing height in both groups (by 0.6 ± 0.3 cm and 0.6 ± 0.4 cm, respectively, *p* = 0.001), with no difference between groups (*p* = 0.777). All other anthropometric variables remained unchanged after training (*p* > 0.5).

### 3.2. Performance Variables

There was no statistical difference between groups in all baseline values (*p* ˃ 0.3; Table 3). No group × time interaction was shown for all other parameters examining various forms of jumping performance (SJ, CMJ, DJ, SLJ, CT, RSI), as well as for the 5 + 5 COD test (Table 3). For all these parameters, except for CT, a main effect for time was observed (*p* < 0.01, η^2^ > 0.07), indicating similar improvements in both groups. However, the improvement in single- and double-leg CMJ in PG had a greater effect size than that in CG (0.72 and 0.67 in PG vs. 0.34 and 0.18 in CG, Table 3). Comparison of the changes in performance between the PG and CG showed a small to moderate effect size, favoring greater improvement of one- and two-leg CMJ in the PG (Table 3). This was also indicated by the MBI analysis that showed a greater likelihood of improvement for the PG (96% and 69% for one- and two-leg CMJ, respectively; see Table 3).

A significant group × time interaction was observed for the 10 and 20 m sprint times (Table 3), with the post hoc test showing an improvement in sprint time only for the PG (*p* = 0.001, ES = 1.10 and 1.14 for 10 and 20 m sprint times, respectively). A significant group × time interaction was also observed for the 10 + 10 m COD test (Table 3), and the post hoc test showed an improvement in the 10 + 10 m COD time only for the PG group (*p* = 0.003, ES = 0.60). This pattern between groups in sprint and COD performance was also evident in effect sizes and quantitative inference analyses (Table 3). Effect size of the change in sprint and COD performance in PG was smaller for the shorter distances examined (i.e., 10 m sprint and 5 + 5 m COD) and 2–3-fold higher for the longer distances (i.e., 20 m sprint and 10 + 10 m COD, see Table 3).

## 4. Discussion

The main findings of this study were that an 8-week supplementary plyometric training program was effective in increasing sprint and COD performance. DJ, SJ, and SLJ were similarly improved in both groups, suggesting that plyometric training did not cause an additional improvement of these types of jumps. However, at the end of the intervention, a greater effect size of the change in single- and double-leg CMJ performance was observed for the PG only.

To the authors’ knowledge, this is the first study to examine the effect of plyometric training on jumping, sprinting, and change of direction abilities in an athletic female population of a very young age. Previous studies in physical education settings involved untrained children, aged 7–11 years, using a form of plyometric training during physical education lessons [22,23]. Findings from these studies are not comparable to an athletic context because physiological adaptations and motor performance differ between non-athletic and athletic youth [15]. Studies in older preadolescent male athletes reported significant increases in horizontal and vertical jumping, as well as in sprint and COD abilities, supporting the findings of the present study [8,24,25,26].

The present study showed that both groups of gymnasts increased SJ, DJ, SLJ, and RSI performance from their respective baselines (main effect time: *p* = 0.01), suggesting that the supplementary plyometric training did not offer additional improvements compared to gymnastics training, following a short training period. A possible explanation of these results is that the gymnastics training program followed by these athletes included a large number of elements with a plyometric component (100 to 130 per training) and, thus, the effect of a short-term supplementary plyometric training period may not be strong enough or may require a longer period to manifest. However, the change in single- and double-leg CMJ performance in the PG had a greater effect size (0.67 and 0.72) and likelihood of improvement (69% and 96%) than the CG (effect size: 0.18 and 0.37), despite the fact that the *p* value for the group × time interaction was not significant (*p* = 0.166 and 0.277, respectively). These effect sizes are in agreement with a recent meta-analysis reporting a larger effect size for younger (<15 years old) compared with older (15–18 years old) girls (0.78 vs. 0.31, respectively) regarding vertical jump improvements following plyometric training [14]. The greater effect size of change in CMJ performance for the PG may be due to the contents of the plyometric training program (Table 2), that contained mostly vertical jumps with a relatively slow stretch–shortening cycle, due to the fact that it was implemented during the preseason. Thus, the neuromuscular and motor learning adaptations may have been more specific for CMJ than the other types of faster stretch–shortening cycle jumps (i.e., DJ). An improvement of CMJ has been reported for older (10–12 years) male child soccer players after 12 weeks of plyometric training in addition to regular soccer training and for 8–12-year-old male pre-PHV hockey players following 6 weeks of a low-dose plyometric training program (60 foot contacts) [11,27].

The results of the present study showed an improvement in 10 and 20 m sprint performance only in the PG, highlighting the importance of complementary plyometric training to improve speed in childhood [28]. Also, plyometric training enhanced 10 + 10m COD only in the PG, while a moderate effect size was also found in 5 + 5 m COD. COD performance reflects a wide range of neuromuscular and locomotor abilities, such as dynamic balance, whole body coordination, speed, as well as eccentric, concentric, and reactive strength [29,30]. For this reason, COD tests have been used as part of the Gymnastics Functional Measurement Tool (GFMT) designed to assess physical fitness in female competitive gymnasts [31], while high correlations have been reported between COD and eccentric strength scaled per kg body mass [32]. A recent review showed that plyometric training improved COD (d = 0.86) in male youths (10–18 years), independent of age [12]. However, greater gains were observed in boys at mid (13–15.9 years, d = 0.95) and post-PHV (16–18 years, d = 0.99) compared to boys at pre-PHV (10–12.9 years, d = 0.68) [12]. Although it seems that the ability to accelerate, decelerate, and change direction is developed at later stages of maturation, the present study showed that COD can be improved following plyometric training in child female athletes, possibly due to an increase in eccentric strength, which is a major determinant of COD performance [32]. The improvement seen in sprint and COD performance may be explained by the reported ‘window of trainability’ for the development of acceleration and maximal velocity during the period between 5 and 9 years [33]. Furthermore, this age is also suggested as an ‘agility, quickness, and COD window’ despite moderate increases in muscular strength and power [1]. Thus, the improvements in sprint and COD performance are possibly related to the high neural plasticity and the increased motor coordination at this stage of development [34]. Also, the training protocol used in this study included a large number of unilateral jumps which induced a marked increase in the sum of the right and left leg CMJ height in the PG compared to the GC. It may be speculated that training using unilateral jumps may be an efficient means for improving sprinting performance and COD, since sprinting and COD involve single-leg actions in successive repetitions. The lack of improvement in sprint performance in the CG indicates that gymnastics training alone is not sufficient to improve sprinting performance. It should be noted that gains in linear sprint and COD were greater in the longer than in the shorter distances. In particular, the effect size for 20 m sprint performance and 10 + 10 m COD was 2–3 times higher than the 10 m sprint and 5 + 5 m COD. One previous study also reported an increase in 0–20 m sprint speed while 0–10 m sprint speed remained unchanged (*p* ˃ 0.05) following 10 weeks of plyometric training in untrained prepubertal boys [35].

This study has some limitations. Despite the considerable improvement of speed and power, the 8-week training period may be considered too short to induce significant differences in some forms of jumping performance (e.g., DJ) in trained young athletes engaged in systematic gymnastics training. Therefore, further studies considering longer training interventions are needed to examine longer-term plyometric training in similar populations. Also, the characteristics of the training program were shaped by the training period (preparation), and were thus generally moderate in intensity and stretch–shortening cycle speed. A progressive continuation of the supplementary plyometric program later in the season, with exercises of higher plyometric load may be more effective. It should be noted that during the intervention period, no injuries were observed, and thus, plyometric training may be considered safe for trained girls, under the condition that the difficulty progression guidelines are followed. 

## 5. Conclusions

In conclusion, the present study showed that plyometric training resulted in a substantial improvement of sprint and COD performance, following 8 weeks of training, in these child female gymnasts. DJ, SJ, and SLJ were similarly improved in both groups, suggesting that additional plyometric training did not result in further increase in these types of jumps during this relatively short timeframe. However, there was a greater effect size of the change in single- and double-leg CMJ performance for the PG only, possibly due to the plyometric training program implemented in this study. Improved CMJ performance may enhance gymnastics skills execution by increasing flight time. Coaches may safely use appropriate supplementary plyometric exercise programs in order to enhance sprinting and change of direction abilities, so as to develop general levels of athleticism in addition to sport-specific physical qualities, in 7–9-year-old female gymnasts. This may have positive implications for long-term athlete development.

## Figures and Tables

**Table 1:** Age, training experience, maturity offset, and anthropometric characteristics of the participants in the plyometric training group (PG) and the control group (CG) (mean ± SD).

Variables	PG (*n* = 33)	CG (*n* = 17)	*p*
Age (year)	8.1 ± 0.7	7.9 ± 0.8	0.333
Training experience (year)	2.5 ± 0.6	2.3 ± 0.5	0.140
Height (cm)	129.3 ± 6.1	129.8 ± 7.6	0.829
Body mass (kg)	28.7 ± 5.8	27.5 ± 6.0	0.475
BMI (kg/m^2^)	17.1± 2.5	16.3 ± 2.0	0.194
Leg length (cm)	60.1 ± 4.1	61.2 ± 4.7	0.420

**Table 2:** Plyometric training program executed two times per week for eight weeks by the athletes in the plyometric training group (PG). Athletes performed two rounds of six exercises in a circuit form, with 30 s rest between exercises and 5 min of rest between rounds.

Weeks: 1 to 4 (Total: 180 Jumps Per Session)	Weeks: 5 to 8 (Total: 190 Jumps Per Session)
1. Rolling squat to jump: standing up on the floor, rolling backward and forward on a 10 cm mat to jump up (10 repetitions)	1. Rolling squat to jump: standing up on the floor, rolling backward and forward on a 10 cm mat to jump up (15 repetitions)
2. Double-leg rope skipping (30 repetitions)	2. Single-leg rope skipping (15 repetitions per leg)
3. Jumping lunges with hands against the wall (6 repetitions per leg)	3. Jumping lunges with hands against the wall (10 repetitions per leg)
4. Depth jumps from one 20 cm box to another 20 cm box (6 repetitions)	4. Depth jumps from one 20 cm box to another 20 cm box (8 repetitions)
5. Box jumps (to a 20 cm box) and step down (12 repetitions)	5. Box jumps (to a 30 cm box) and step down (12 repetitions)
6. Lateral double-leg line jumps (20 repetitions)	6. Lateral double-leg line jumps to a half-squat position (10 repetitions)

**Table 3:** Changes in performance variables following the 8 weeks of intervention for the plyometric training group (PG, *n* = 33) and the control group (CG, *n* = 17).

Variables		Pre-Training	Post- Training	Cohen’s d (Pre vs. Post-Training)	Change from Pre- to Post-Training (90% CI)	Cohen’s *d* and Quantitative Inference PG vs. CG (PG, Trivial, CG)	*p* Value Group × Time Interaction
CMJ (cm)	PG	18.1 ± 3.2	20.1 ± 2.9	0.67	2.0 (1.2 to 2.8)	0.48	0.166
	CG	17.0 ± 4.5	17.8 ± 4.6	0.18	0.8 (−0.4 to 2.0)	(69%, 30%, 1%)	
R + L CMJ (cm)	PG	15.2 ± 4.2	18.1 ± 4.0	0.72	3.0 (1.7 to 4.3)	0.34	0.277
	CG	14.3 ± 5.1	15.9 ± 4.7	0.34	1.6 (0.3 to 2.9)	(96%, 4%, 0%)	
DJ (20 cm) (cm)	PG	15.3 ± 3.9	17.3 ± 3.3	0.56	2.0 (1.0 to 3.0)	0.15	0.634
	CG	13.9 ± 4.3	16.4 ± 4.1	0.61	2.5 (1.1 to 3.9)	(44%, 45%, 11%)	
SJ (cm)	PG	17.3 ± 3.4	19.0 ± 3.2	0.52	1.7 (1.0 to 2.4)	0.18	0.547
	CG	16.3 ± 4.0	17.5 ± 3.5	0.33	1.2 (−0.2 to 2.6)	(76%, 22%, 2%)	
SLJ (cm)	PG	112.5 ± 14.3	134.7 ± 14.5	1.57	22.2 (17.5 to 26.9)	0.20	0.507
	CG	107.4 ± 17.9	126.6 ± 17.7	1.11	19.3 (14.9 to 23.7)	(25%, 52%, 23%)	
COD 5 + 5 m (s)	PG	3.77 ± 0.36	3.54 ± 0.28	0.72	−0.24 (‒0.33 to −0.15)	0.39	0.203
	CG	3.81 ± 0.37	3.71 ± 0.28	0.31	−0.11 (−0.27 to 0.05)	(59%, 32%, 9%)	
COD 10 + 10 m (s)	PG	5.94 ± 0.42	5.71 ± 0.36 *	0.60	−0.22 (−0.32 to −0.12)	1.00	0.008
	CG	6.02 ± 0.47	6.14 ± 0.48	0.26	0.11 (−0.01 to 0.23)	(98%, 2%, 0%)	
10 m sprint (s)	PG	2.77 ± 0.26	2.51 ± 0.22 *	1.10	−0.26 (−0.33 to −0.19)	0.40	0.018
	CG	2.74 ± 0.24	2.65 ± 0.25	0.38	−0.10 (−0.04 to −0.16)	(86%, 13%, 1%)	
20 m sprint (s)	PG	4.85 ± 0.54	4.36 ± 0.30 *	1.14	−0.50 (−0.64 to −0.36)	0.81	0.011
	CG	4.78 ± 0.45	4.62 ± 0.37	0.40	−0.16 (−0.27 to −0.05)	(97%, 3%, 0%)	
CT (ms)	PG	306 ± 77	301 ± 84	0.05	−4 (−38 to 29)	0.14	0.642
	CG	282 ± 60	290 ± 73	0.12	8 (−22 to 38)	(90%, 9%, 1%)	
RSI (mm/ms)	PG	0.57 ± 0.22	0.65 ± 0.22	0.37	0.08 (0.02 to 0.14)	0.00	0.998
	CG	0.55 ± 0.21	0.63 ± 0.24	0.37	0.08 (−0.04 to 0.20)	(85%, 14%, 1%)	

* *p* < 0.01 from the corresponding pre-training value; CMJ: counter movement jump; R + L CMJ: sum of right and the left leg counter movement jumps; DJ: drop jump; SJ: squat jump; SLJ: standing long jump; COD 5 + 5 m: 5 + 5 m change of direction test with a 180° turn; COD 10 + 10 m: 10 + 10 m change of direction test with a 180° turn.

## References

[1] Lloyd R.S., Oliver J.L. (2012). The youth physical development model: A new approach to long-term athletic development. Strength Cond. J..

[2] Pichardo A.W., Oliver J.L., Harrison C.B., Maulder P.S., Lloyd R.S. (2018). Integrating models of long-term athletic development to maximize the physical development of youth. Int. J. Sports Sci. Coach..

[3] Granacher U., Lesinski M., Büsch D. (2016). Effects of Resistance Training in Youth Athletes on Muscular Fitness and Athletic Performance: A Conceptual Model for Long-Term Athlete Development. Front. Physiol..

[4] Meyers R.W., Oliver J.L., Hughes M.G., Lloyd R.S., Cronin J.B. (2017). New insights into the development of maximal sprint speed performance in male youth. Strength Cond. J..

[5] Lloyd R.S., Read P., Oliver J.L., Meyers R.W., Nimphius S., Jeffreys I. (2013). Considerations for the development of agility during childhood and adolescence. Strength Cond. J..

[6] Moeskops S., Oliver J.L., Read P.J., Cronin J.B., Myer G.D., Lloyd R.S. (2018). The Physiological Demands of Youth Artistic Gymnastics; Applications to Strength and Conditioning. Strength Cond. J..

[7] Ramirez-Campillo R., Álvarez C., García-Hermoso A. (2018). Methodological Characteristics and Future Directions for Plyometric Jump Training Research: A Scoping Review. Sport Med..

[8] Michailidis Y., Fatouros I.G., Primpa E. (2013). Plyometrics trainability in preadolescent soccer athletes. J. Strength Cond. Res..

[9] Radnor J.M., Oliver J.L., Waugh C.M., Myer G.D., Moore I.S., Lloyd R.S. (2018). The Influence of Growth and Maturation on Stretch-Shortening Cycle Function in Youth. Sports Med..

[10] Lesinski M., Prieske O., Granacher U. (2016). Effects and dose-response relationships of resistance training on physical performance in youth athletes: A systematic review and meta-analysis. Br. J. Sports Med..

[11] Moran J.J., Sandercock G.R.H., Ramírez-Campillo R., Meylan C.M.P., Collison J.A., Parry D.A. (2017). Age-related variation in male youth athletes’ countermovement jump after plyometric training: A meta-analysis of controlled trials. J. Strength Cond. Res..

[12] Asadi A., Arazi H., Ramirez-Campillo R., Moran J., Izquierdo M. (2017). Influence of Maturation Stage on Agility Performance Gains after Plyometric Training: A Systematic Review and Meta-analysis. J. Strength Cond. Res..

[13] Boisseau N., Delamarche P. (2000). Metabolic and hormonal responses to exercise in children and adolescents. Sport Med..

[14] Moran J., Clark C.C.T., Ramirez-Campillo R., Davies M.J., Drury B. (2018). A Meta-Analysis of Plyometric Training in Female Youth. J. Strength Cond. Res..

[15] Faigenbaum A.D., Myer G.D. (2010). Pediatric resistance training: benefits, concerns, and program design considerations. Curr. Sports Med. Rep..

[16] Faigenbaum A.D., Myer G.D., Farrell A. (2014). Integrative neuromuscular training and sex-specific fitness performance in 7-year-old children: An exploratory investigation. J. Athl. Train..

[17] Mirwald R.L.G., Baxter-Jones A.D., Bailey D.A., Beunen G.P. (2002). An assessment of maturity from anthropometric measurements. Med. Sci. Sport Exerc..

[18] Glatthorn J.F., Gouge S., Nussbaumer S., Stauffacher S., Impellizzeri F.M., Maffiuletti N.A. (2001). Validity and reliability of Optojump photoelectric cells for estimating vertical jump height. J. Strength Cond. Res..

[19] Flanagan E.P., Comyns T.M. (2008). The use of contact time and the reactive strength index to optimize fast stretch-shortening cycle training. Strength Cond. J..

[20] Lloyd R.S., Meyers R.W., Oliver J.L. (2011). The Natural Development and Trainability of Plyometric Ability During Childhood. Strength Cond. J..

[21] Hopkins W.G., Marshall S.W., Batterham A.M., Hanin J. (2009). Progressive statistics for studies in sports medicine and exercise science. Med. Sci. Sports Exerc..

[22] Arabatzi F., Tziagkalou E., Kannas T., Giagkazoglou P., Kofotolis N., Kellis E. (2018). Effects of two plyometric protocols at different surfaces on mechanical properties of achilles tendon in children. Asian J. Sports Med..

[23] Faigenbaum A.D., Farrell A.C., Radler T. (2009). “Plyo Play”: A Novel Program of Short Bouts of Moderate and High Intensity Exercise Improves Physical Fitness in Elementary School Children. Phys. Educ..

[24] Chaabene H., Negra Y. (2017). The effect of plyometric training volume on athletic performance in prepubertal male soccer players. Int. J. Sports Physiol. Perform..

[25] Bouguezzi R., Chaabene H., Negra Y. (2018). Effects of Different Plyometric Training Frequency on Measures of Athletic Performance in Prepuberal Male Soccer Players. J. Strength Cond. Res..

[26] Lloyd R.S., Radnor J.M., De Ste Croix M.B.A., Cronin J.B., Oliver J.L. (2016). Changes in Sprint and Jump Performances After Traditional, Plyometric, and Combined Resistance Training in Male Youth Pre- and Post-Peak Height Velocity. J. Strength Cond. Res..

[27] Moran J., Sandercock G.R.H., Ramírez-Campillo R., Todd O., Collison J., Parry D.A. (2017). Maturation-Related Effect of Low-Dose Plyometric Training on Performance in Youth Hockey Players. Pediatr. Exerc. Sci..

[28] Behm D.G., Young J.D., Whitten J.H.D. (2017). Effectiveness of traditional strength vs. power training on muscle strength, power and speed with youth: A systematic review and meta-analysis. Front. Physiol..

[29] Brughelli M., Cronin J., Levin G., Chaouachi A. (2008). Understanding change of direction ability in sport. Sports Med..

[30] Hader K., Palazzi D., Buchheit M. (2015). Change of direction speed in soccer: How much braking is enough?. Kinesiology.

[31] Sleeper M.D., Kenyon L.K., Casey E. (2012). Measuring fitness in female gymnasts: The gymnastics functional measurement tool. Int. J. Sports Phys. Ther..

[32] Spiteri T., Nimphius S., Hart N.H., Specos C., Sheppard J.M., Newton R.U. (2014). Contribution of Strength Characteristics to Change of Direction and Agility Performance in Female Basketball Athletes. J. Strength Cond. Res..

[33] Rumpf M.C., Cronin J.B., Pinder S.D., Oliver J., Hughes M. (2012). Effect of Different Training Methods on Running Sprint Times in Male Youth. Pediatr. Exerc. Sci..

[34] Branta C., Haubenstricker J., Seefeldt V. (1984). Age changes in motor skills during childhood and adolescence. Exerc. Sport Sci. Rev..

[35] Kotzamanidis C. (2006). Effect of plyometric training on running performance and vertical jumping in prepubertal boys. J. Strength Cond. Res..

